# Renal Oncocytoma: A Systematic Review of Its Metastatic Features

**DOI:** 10.7759/cureus.71649

**Published:** 2024-10-16

**Authors:** Ragaul Rajagopal, Edzhem Yoztyurk, Kapilraj Ravendran

**Affiliations:** 1 Urology, East Sussex Healthcare NHS Trust, Eastbourne, GBR; 2 Urology, Gradscape, London, GBR; 3 Internal Medicine, Medical University Sofia, Sofia, BGR; 4 Internal Medicine, Gradscape, London, GBR; 5 Rheumatology, Royal National Orthopaedic Hospital, London, GBR; 6 Surgery, Gradscape, London, GBR

**Keywords:** cancer, histological features, metastatsis, renal oncocytoma, urologic oncology

## Abstract

Oncocytomas are referred to as benign kidney neoplasms. They primarily affect adults, with patients over 70 years old being the most affected. Renal oncocytomas (ROs) are frequently detected by excision, biopsy, or scan. Hematuria, flank pain, and a palpable mass are the traditional trio of symptoms. Oncocytomas appear as well-circumscribed, tan or mahogany-coloured masses with a central scar that is stellate. Histological features include well-circumscribed lesions, bland cytology, eosinophilic cytoplasm, regular nuclei with prominent central nucleoli, and nested architecture. ROs are rarely linked to an aggressive clinical course and have an excellent prognosis. There is proof that the disease can spread to the liver and bones. Some literature has also reported oncocytoma metastases to the lung and liver. This systematic review of the literature examines and evaluates the malignant potential of oncocytoma. The purpose of the study was to determine whether ROs can be diagnosed as a benign condition or if malignancy needs to be considered and investigated. Seventeen studies were analysed which had a total of 412 ROs. Four patients (one percent) died as a result of their illness. There was evidence of disease progression in every patient who passed away from their illness. Six patients (1.5%) experienced disease progression in total. Three hundred and seventeen patients (80%) underwent radical nephrectomy, while 81 patients (20%) underwent partial nephrectomy. Liver, bone, lung, lymphadenopathy, and local recurrence were among the metastasis sites. Perinephric fat invasion, renal sinus fat invasion, renal capsular invasion, and vascular invasion are characteristics of metastatic behavior that have been found. Despite this, the small number of patients who experienced disease progression and/or death as a result of ROs implies that aggressive malignant behavior is not always correlated with the presence of metastatic features or disease. Oncocytomas should be viewed as having a low potential for malignancy rather than as benign. Individuals who exhibit aggressive characteristics, such as vascular invasion and/or perinephric fat invasion, have an atypically good prognosis. Despite advancements in imaging and immunochemical techniques, it is indisputable that ROs, which were first classified as renal tumours in 1976, continue to pose a diagnostic challenge for multidisciplinary teams. There is considerable variation in practice across the globe due to difficulties in confirming ROs, especially when it comes to metastatic disease. There is even more variation in the management of follow-up care that follows. This will remain the MDT's current state until randomised controlled trials, long-term results, and a better comprehension of the behavior of this tumour are obtained.

## Introduction and background

Renal oncocytomas (ROs) account for around 3-8% of renal cancers and referred benign kidney neoplasms [1]. They appear mainly in adults, most commonly in patients over the age of 70. ROs are often diagnosed via biopsy, scan, or excision. A classical triad of symptoms includes palpable mass, hematuria, and flank pain [2]. Oncocytomas on gross appearance are well-circumscribed tan or mahogany-coloured masses with a stellate central scar [3]. They histologically show nested architecture, regular nuclei with prominent central nucleoli, bland cytology, eosinophilic cytoplasm, and well-circumscribed lesions [3]. The classic histologic appearance of ROs is nests and tubular structures lined by eosinophilic, granular cytoplasm cells. Oncocytomas usually show edematous myxoid or hyalinized stroma, that can result in nests and tubular formations scattered throughout the stroma [3]. Additionally, there may be isolated instances of clear cytoplasm, usually around the central scar. The nuclei exhibit regularity and roundness [3].

Using immunohistochemistry and specific stains, such as Hale's colloidal iron stain can help with the differential diagnosis of ROs and other oncocytic neoplasms by providing valuable diagnostic criteria [3]. Cytokeratin 7 (CK7) staining is typically very minimal in oncocytomas, whereas renal cell carcinoma can be diffusely positive in a membranous distribution [3]. In a recent study, the most widely used staining method for oncocytoma diagnosis among urologic pathologists was CK7 [3]. Some laboratories use other markers, such as kidney-specific cadherin and S100A1, though these are not as commonly used [3]. Since both oncocytoma and chromophobe renal cell carcinoma share frequent membranous positivity for KIT and negative staining for vimentin, markers like KIT (CD117) and vimentin may be useful for differentiating between these two subtypes of renal cell carcinoma from others, such as clear cell and papillary renal cell carcinoma variants with eosinophilic cytoplasm [3].

There are, however, many types that mimic oncocytoma and therefore can be challenging. Diagnosis is primarily made using histology and in challenging cases, with immunohistochemistry [3].

ROs are treated conservatively with partial nephrectomy depending on the size of the tumor. There are occasions where oncocytomas are treated with a total nephrectomy [4]. An alternative treatment option is tumor ablation which has shown to be more cost-effective [5]. Thermoablative treatment consists of percutaneous or surgical cryoablation and radiofrequency ablation [6]. It is important to recognize the potential pitfalls that may cause doubt about the diagnosis, for example, renal fat involvement, vascular invasion, and degenerative atypia [4]. As renal mass biopsies are being used more frequently, it's critical to understand the technique's limitations and apply the right standards when making a diagnosis of oncocytoma in a small biopsy specimen. [4].

Recent studies showed that biopsy-proven ROs are suitable for active surveillance and suggest that it is safe to monitor [7]. Active surveillance includes monitoring the size of the tumour, and a CT scan is the most commonly used imaging technique in the follow-up [8].

ROs have an excellent prognosis and are rarely associated with an aggressive clinical course. There has been evidence that there can be metastasis to the liver and bone [4]. Metastasis of oncocytomas to the liver and lung have also been reported in some literature [9].

This is a systematic review to see and analyze the malignancy potential of oncocytoma. The aim was to establish whether oncocytoma should be described as benign or whether malignancy should be taken into account and followed up when making a diagnosis of ROs. A collection of studies were analyzed and the findings are summarized.

## Review

Methodology

Search Strategy and Inclusion Criteria

This systematic review assessed the advantages, justification, and malignancy evidence for ROs.

The PRISMA (Preferred Reporting Items for Systematic Reviews and Meta-analyses) guidelines [10] and the protocol, which was developed collaboratively and approved by all authors, were followed for conducting this review. Bias risk was assessed, and disagreements regarding bias and the best way to interpret the data were resolved by consensus-building talks.

A literature search was carried out using the following keywords: "renal oncocytoma," "metastases," and "metastatic" via MEDLINE (available through PubMed), Scopus, and Google Scholar: Only English-language publications were included in the search.

Study Selection

Oncocytoma malignancy evidence was one of the review's inclusion requirements. Also noted as secondary outcomes were the extent of malignancy and the course of the illness. Exclusion criteria were met by studies whose writing was not in English and whose texts could not be viewed in their entirety online. Randomized controlled trials, prospective and retrospective research, case reports, and case studies were the only types of literature in our review. Not included were editorials or systematic reviews.

For the first screening, the titles and abstracts of two researchers (R.R. and E.Y.) were used. In terms of evaluating bias and interpreting results, one investigator (K.R.) resolved their disagreements. Full-text analysis was conducted on abstracts that had incomplete information. After that, the same researchers evaluated each full-text article's eligibility on their own. Disputes over the assessment of bias and the interpretation of the results were resolved through consensus discussions. Once the articles were reviewed, their full texts were acquired and expunged if it was determined that they had nothing to do with the evaluation subject.

Data Extraction and Quality Assessment

One senior researcher (K.R.) reviewed the results after two researchers (R.R. and E.Y.) extracted the data using standardized criteria. The following details were taken out of the data: journal, year of publication, databases searched, length of time, number of studies, total patients and countries, study design, outcomes, main conclusions, main limitations, and implications: opportunities and difficulties.

Each study's quality evaluation and risk of bias were independently assessed by two authors (R.R., K.R.) using the Risk Of Bias In Non-randomized Studies-of Interventions (ROBINS-I) tool [11]. This is a quantitative method for measuring the efficacy of treatments, not randomized research. It evaluates confounding bias, bias in the process of selecting research participants, bias in the categorization of the interventions, bias in the case that planned interventions diverge from reality, prejudice in the evaluation of results, and bias in the selection of the published result. For each article, a quality evaluation summary was created using the Risk of Bias Visualization (ROBVIS) tool [12].

After looking through the included articles, details regarding our study's conclusions were taken out. In this review, the PRISMA guidelines adhered to the study by Page et al. [10].

Results

**Figure 1:**
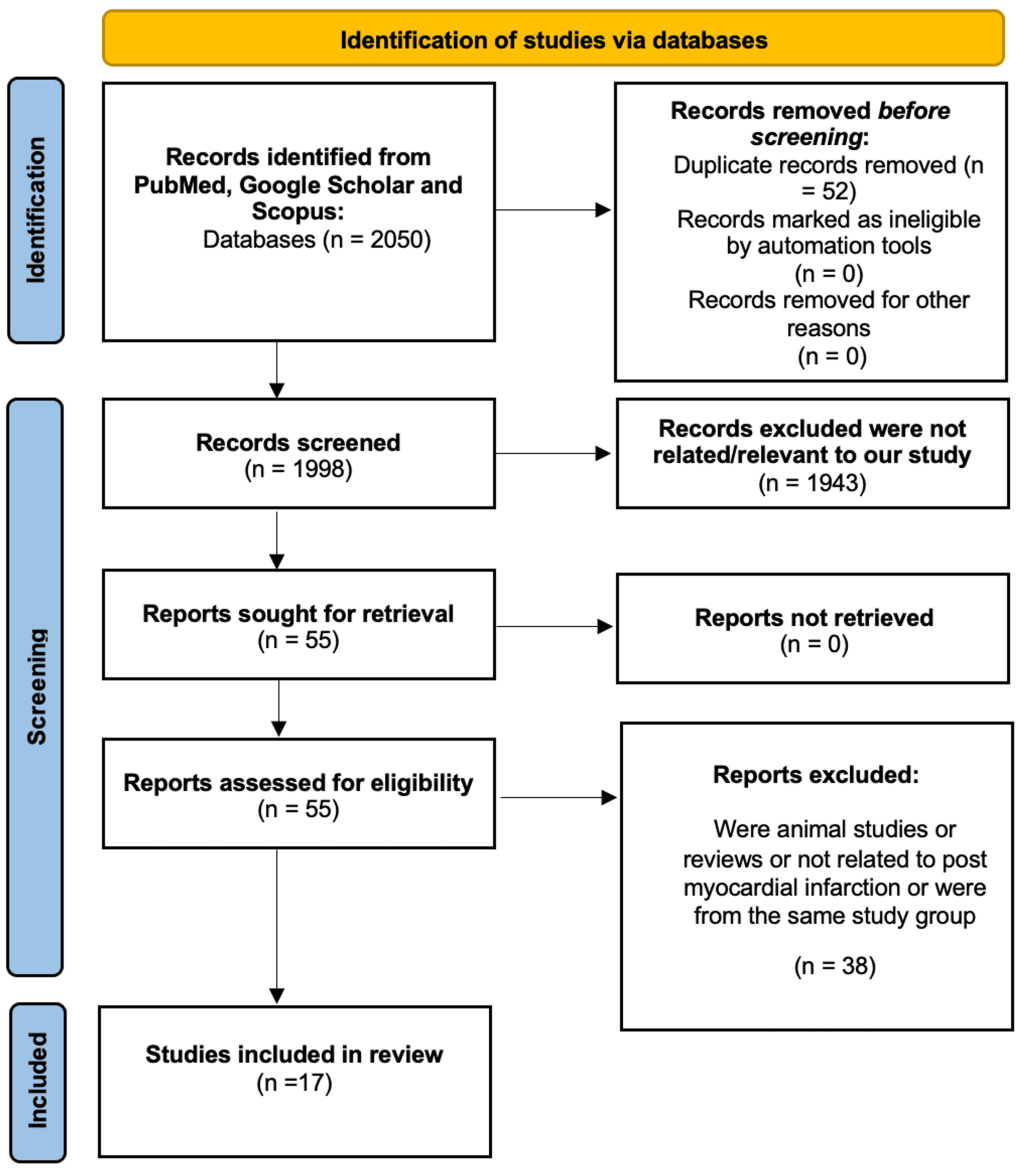
PRISMA flow diagram demonstrating the literature selection strategy. PRISMA: Preferred Reporting Items for Systematic Reviews and Meta-Analyses

Figure 1 shows and demonstrates our search and articles examined as shown in the PRISMA diagram [10].

From the 17 papers, a total of 412 patients with RO were reported [2,9,13-27]. Gudbjartsson et al. reported 45 patients; however, 14 of these had an incidental autopsy biopsy-proven RO for an unrelated disease and were therefore not included in this literature review analysis [13], resulting in a total of 398 patients. Each study was analysed with the following criteria: patient demographics, tumour characteristics, metastatic feature and or site, mean follow-up, management, disease progression, and death secondary to disease. Table 1 provides a summary of the 17 papers included in this systematic review.

**Table 1:** Summary of publications included in this literature review. *: Fourteen of the 45 patients had incidental autopsy biopsy-proven RO for an unrelated disease and therefore have been excluded; ** Re-presented nine years following discharge; ₽: 19 patients were lost to follow up; R: Right; L: Left; ≠: Had transition to high-grade oncocytic oncocytoma; †: Six patients had no follow-up data, including the patient with biopsy-proven oncocytoma; ‡: No follow-up information for two patients; ≥: This includes multifocal unilateral and bilateral RO. NA: Not applicable; VI: Vascular invasion; PNFI: Perinephric fat invasion; RSFI: Renal sinus fat invasion; CI: Renal capsular invasion; PN: Partial nephrectomy; RN: Radical nephrectomy; RO: Renal oncocytoma

Study	Study Design	Patients with RO (n=)	Age, mean (range)	Gender: Male N (%)	Side: Right N (%)	Bilateral N (%)	Size mm (range)	Metastatic feature and or site N (%)	Follow-up mean months (range)	Management (n=)	Disease progression (n=)	Death secondary to disease (n=)
Psihramis et al. (1988) [14]	Case Report	1	73 (NA)	1 (100)	NA	1 (100)	R 55 L 105	VI, PNFI, CI 1	6 (NA)	R PN L RN	0	0
Amin et al. (1997) [27]	Retrospective	80	67.2 (32-89)	NA	NA	4 (4.3)	44 (60-150)	VI 12 (15%) PNFI 9 (11%) CI 8 (10%)	91.2 (15-200)	12 PN 68 RN	0	0
Perez-Ordonez et al. (1997) [15]	Comparative Study	70	65 (25-86)	39 (56)	35 (50)	3 (4)	52 (14-140)	VI 3 (4%) PNFI 14 (20%) Liver 1 Liver and bone 1	58 (1-181)	9 PN 61 RN	1	1
Amin et al. (1999) [2]	Case report	1	48 (NA)	1 (100)	0 (0)	0 (0)	150 (NA)	Bone 1	24 (NA)	1 RN	1	1
Gudbjartsson et al. (2005) [13]	Retrospective	45*	70.5 (41-91)	18 (58)	17 (55)	1 (3.2)	57 (9-120)	CI 1 (3 %) PNFI 2 (6%)	99.6 (NA)	30 RN 1 PN	0	0
Oxley et al. (2007) [16]	Case Report	1	70’s (NA)	0 (0)	0 (0)	0 (0)	120 (NA)	Liver 1	18 (NA)	1 RN	1**	1
Hes et al. (2008) [17]	Retrospective	7	72.9 (61-82)	5 (71)	3 (42)	0 (0)	54 (22-75)	VI 7 (100%)	43.2 (12-60)	7 RN	0	0
Trpkov et al. (2009) [18]	Retrospective	109	63.5 (36-91)			4.6	40.3 (10.5-160)	VI 4 (3.7%) PNFI 17 (15.6%)	52 (1-113)	82 RN 27 PN	0^₽^	0
Trivedi et al. (2013) [19]	Case Report	1	48 (NA)	0 (0)	0 (0)	0 (0)	10 (NA)	Liver 1	Ongoing	1 RN	1	0
Kolníková et al. (2014) [20]	Case Report	1	69 (NA)	1 (100)	1 (100)	0 (0)	90 (NA)	PNFI 1	12	1 RN	0	0
Sirintrapun et al. (2014) [21]	Case Report	1^≠^	74 (NA)	1 (100)	1 (100)	0 (0)	110 (NA)	Lymphadenopathy 1 Lung 1	29	1 RN	1	1
Williamson 2016 [22]	Case Report	1	NA	NA	NA	NA	NA	PNFI 1	NA	1RN	0	0
Wobker et al. (2016) [23]	Case Series	22	67.5 (48-91)	12 (54)	10 (45)	0 (0)	52 (10-120)	VI 22 (100) PNFI 7 (32)	29.9 (7.5-94.5)	5 PN 16 RN 1 Biopsy†	0†	0†
Amante et al. (2017) [24]	Case Report	1	62 (NA)	0 (0)	1 (100)	0 (0)	75 (NA)	CI 1 Local recurrence 1 Bone 1	48 (NA)	1 RN	1	0
Cacciamani et al. (2019) [9]	Case Report	1	62 (0)	0 (0)	0 (0)	0 (0)	79 (NA)	Liver 1	12 (NA)	1RN	0	0
Omiyale and Carton (2019) [25]	Retrospective	20	64.45 (33-88)	70	NA	0	51 (20-135)	VI PNFI	25.6 (2-103)	9 PN 11 RN	0‡	0‡
Al-Obaidy and Cheng (2021) [26]	Case Series	50	68 (50-87)	38 (76)	23 (46)	6 (12)^≥^	53 (15-157)	VI 5 (10) PNFI 25 (50) RSFI 9 (18)	54 (1-144)	17 PN 33 RN	0	0

A total of four patients (1%) reported death secondary to disease; however, Wobker et al. had no follow-up data for six of their patients (30%) [23]. Trpkove et al. lost 19 patients to follow up (17.4%) [18], and Omiyale et al. had no follow-up data for two patients (10%) [25]. All the patients who died from the disease had evidence of disease progression. A total of six patients (1.5%) had disease progression, with Oxley et al. presenting nine years following discharge [16]. Radical nephrectomy was performed on 317 patients (80%) and partial nephrectomy on 81 patients (20%).

Sites of metastasis included liver, bone, lung, lymphadenopathy, and local recurrence. Features of metastatic behaviour identified include perinephric fat invasion, renal sinus fat invasion, renal capsular invasion, and vascular invasion. Despite this, the low number of patients who went on to have disease progression and/or death secondary to RO suggests that the presence of metastatic features or disease does not always equate to aggressive malignant behaviour.

Quality of Assessment

**Figure 2:**
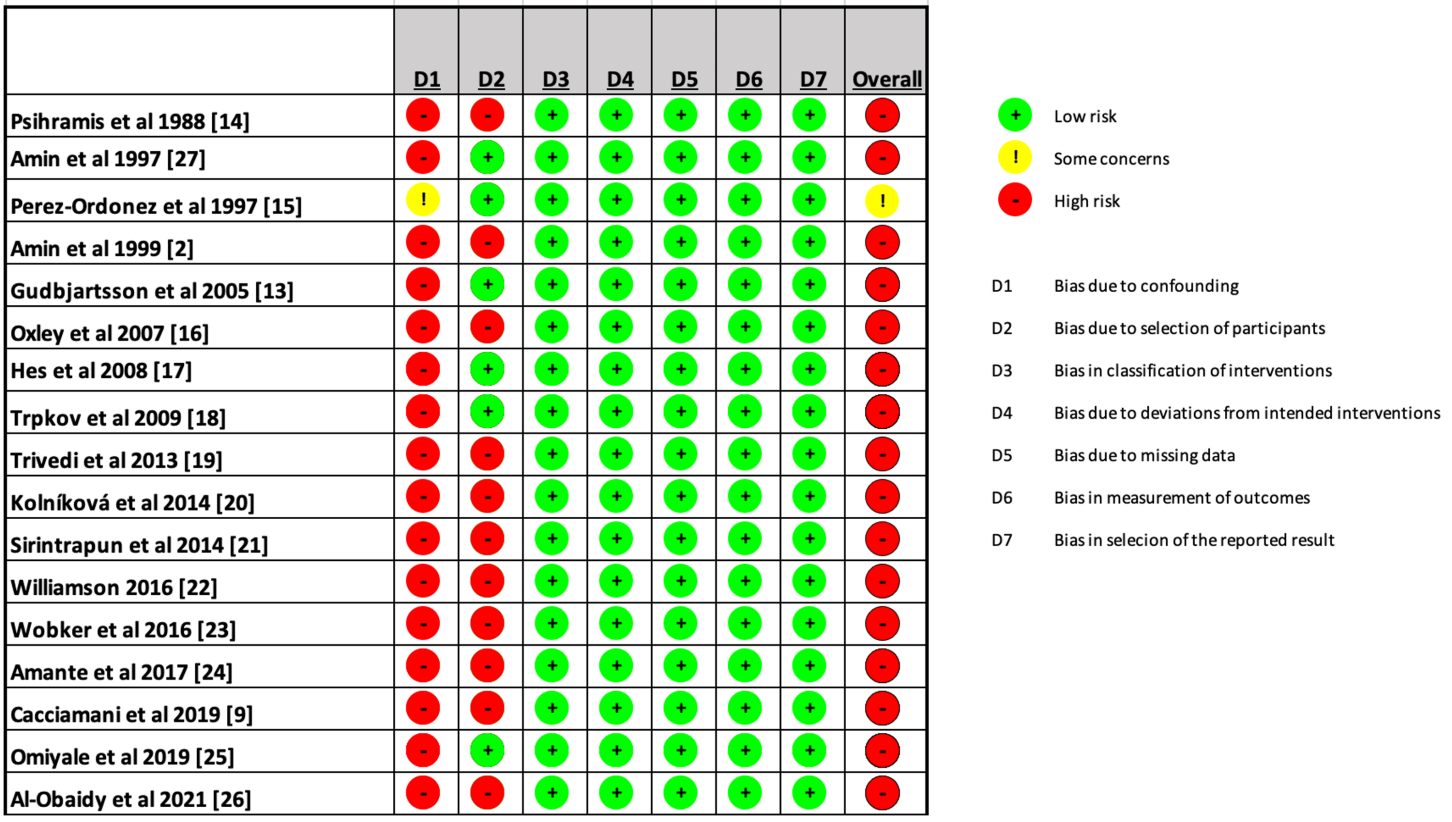
Detailed assessment of every study included in the systematic review using the Risk of Bias Visualization (ROBVIS) tool. Source: [12] D1: Bias due to confounding; D2: Bias due to selection of participants; D3: Bias in classification of interventions; D4: Bias due to deviations from intended interventions; D5: Bias due to missing data; D6: Bias in measurement of outcomes; D7: Bias in selection of the reported result

All of the included studies were classified as having a high risk of bias. Since most of the research are case reports, case studies and retrospective studies, confounding makes them extremely susceptible to bias. Figure 2 presents a detailed assessment of every study included in this systematic review using the ROBVIS tool [12].

Discussion

Histology and Immunochemistry

ROs are often difficult to differentiate from renal cell carcinoma (RCC) due to similarities in clinical presentation, radiological, morphological and at times histological characteristics [28]. Histology and immunochemistry are therefore essential to diagnosing RO.

On gross appearance, ROs are tan or mahogany coloured and resemble normal renal parenchymal tissue. Clear cell RCC is typically golden yellow. ROs can also present with a central stellate scar; however, this feature can also be demonstrated in chromophobe RCC, and in a study of 70 patients, a central stellate scar was only present in 33% of cases [28].

Histologically ROs are well-circumscribed lesions with regular round nuclei that are prominent and have eosinophilic granular appearing cytoplasm [23]. Because they have oedematous myxoid or hyalinized stroma, it can result in nests and tubular-like structures dispersed in the stroma which are lined by these eosinophilic granular cytoplasm cells [23].

Tumours can also be examined at an ultrastructural level. Here the high level of mitochondria is a key identifier in confirming RO and explains why there is an appearance of granularity in microscopy [16].

The use of immunohistochemical staining is key in the differential diagnosis of RO [16]. Malignant and benign epithelial tumours are characterised by which cytokeratins they express [23]. Only 10% of RO express Cytokeratin 7 (CK 7), compared to chromophobe RCC where it is expressed in 73% [28]. This is consistent with our case where only patchy staining for CK 7 was demonstrated. CK 7 is the most common staining technique for diagnosing oncocytomas [23]. When differentiating chromophobe RCC and RO with other RCC with eosinophilic cytoplasm, C-KIT (CD117) stains positively while vimentin is negative. C-KIT was found to be expressed by both RO and Chromophobe RCC but not detected in most subtypes [23].

The World Health Organisation (WHO) classification for Tumours of the Urinary System and Male Genital Organs in 2016 had RO categorised as a benign epithelial renal tumour; however, in its latest update in 2022 there is a new category for “other oncocytic/chromophobe RCC” for tumours with a low metastatic potential demonstrating the need for further classification of these challenging tumours [29].

Perinephric and Renal Vein Involvement

ROs have been reported to display atypical features such as vascular and/or perinephric fat invasion. Extension into veins and perinephric fat indicate oncocytomas have invasive potential [11]; however, the natural history of this disease is still poorly understood. Of the 17 papers included in this literature review, 10 papers described patients with perinephric and or renal vein involvement [13,14,18,20,22,23,25-28]. The study by Omiyale et al. on 159 patients with surgically resected ROs demonstrated that 12.6% had vascular and or perinephric fat invasion [25]. Despite this, no patients had disease progression or death, with them concluding that these features do not alter their favourable prognosis and benign course, with Williamson rendering the same conclusion [22,25].

Distant Metastases 

Of the 398 patients, 5 (1.4%) were reported to have liver metastases. Cacciamani et al. demonstrated that both the liver lesions and renal mass had features consistent with RO, with solid nests of round to polygonal cells associated with densely granular eosinophilic cytoplasm and round regular nuclei [9]. Their immunohistochemistry was identical and positive for S100A, CD10 and CD117. Perez-Ordonez et al. describe two patients with liver metastases, of which one had concomitant bone metastasis [28]. The patient with solely liver metastases had biopsy-confirmed disease performed at the time of the radical nephrectomy. They presented with vascular invasion and pT3 disease, but at 58 months of follow up had stable disease [28]. Oxley et al. discussed a patient who underwent a radical nephrectomy which was histologically proven to be a RO [16]. The patient re-presented nine years later with liver metastasis, which the biopsy confirmed as a RO with well-defined islands of oncocytic cells arranged in nests [16]. Immunohistochemistry was comparable to the original renal tumour, with kidney-specific cadherin (Ksp cadherin), demonstrating 10% marking [16]. Trivedi et al. describe a small cell variant of RO which was managed by radical nephrectomy [19]. The patient developed liver metastases on surveillance imaging 14 months later. Liver biopsy demonstrated normal liver parenchyma with a focus on oncocytic cells which was positive for CD117. The patient was managed on adjuvant therapy (sirolimus) and the disease decreased in size.

Oncocytomas: A Diagnostic Challenge

ROs have always posed a diagnostic dilemma to the multi-disciplinary team. Cross-sectional imaging provides limited diagnostic accuracy; therefore, histological confirmation is deemed the most reliable modality for a definitive diagnosis. Renal mass biopsies (RMBs) are used as a diagnostic tool, however controversially they do not always exclude a malignancy due to the association of hybrid tumours, such as chromophobe renal cell carcinoma and RO tumours [30]. RMBs in the diagnosis of ROs have their limitations which was highlighted by a recent international snapshot study that demonstrated only 29% of clinicians would routinely offer a RMB for a possible RO in a SRM [31]. Studies have reported varying positive predictive values regarding biopsy accuracy with post-operative histology confirmed RO in only 64.6% and 67% of renal tumours [32,33], although conversely one large single-centre study found 94% accuracy [34]. There have however been promising studies with the use of Technetium (99mTc)-sestamibi single-photon emission computed tomography (SPECT) CT in differentiating RO from RCC [35-37] with a prospective trial in the UK currently assessing its applicability for use in the NHS (ISRCTN23705289). As ROs are rich in mitochondria, they have an avid uptake of tracer making the mass ‘hot’ on imaging, whilst RCC actively transport the tracer out of their cells making them ‘cold’ [31].

Four decades ago, high numbers of patients were reported to be dying from metastatic oncocytomas. One study suggested a classification system for Grades 1, 2 and 3. Those diagnosed with ‘grade 2’ were noted to have a high risk of developing metastases [38]. These cases however were re-examined and re-classified as chromophobe RCC [27,28]. A review study reported and analysed 166 ROs with metastatic presentations, which was excluded from this literature review and found that 10.2% and 5.4% of cases demonstrated perinephric fat invasion and vascular invasion, respectively [39]. Another study noted that granular cell carcinoma could have been a differential diagnosis, and the remaining metastatic cases were dismissed as RO on histological review [28].

Limitations

Most of the included research may contain biases because of the nature of the studies included. Future studies should give more detailed information on this important topic.

## Conclusions

Oncocytomas should not be considered benign, but rather have low malignant potential. Patients with aggressive features such as vascular and/or perinephric fat invasion unusually retain a favourable prognosis. Since ROs were established as a renal tumour subclassification in 1976, it remains undeniable that they still present a diagnostic dilemma to the MDT, despite the evolution in imaging and immunochemical techniques. Challenges in confirming RO, particularly in the context of metastatic disease, have resulted in a wide variation in practice internationally, with even more diversity in subsequent follow-up management. Until randomised controlled trials, long-term outcomes and a deeper understanding of this tumour's behaviour, this will continue to be the status quo for the MDT.
